# Publisher Correction: Carbohydrate fatty acid monosulphate: oil-in-water adjuvant enhances SARS-CoV-2 RBD nanoparticle-induced immunogenicity and protection in mice

**DOI:** 10.1038/s41541-023-00634-w

**Published:** 2023-03-03

**Authors:** Etsuro Nanishi, Francesco Borriello, Hyuk-Soo Seo, Timothy R. O’Meara, Marisa E. McGrath, Yoshine Saito, Jing Chen, Joann Diray-Arce, Kijun Song, Andrew Z. Xu, Soumik Barman, Manisha Menon, Danica Dong, Timothy M. Caradonna, Jared Feldman, Blake M. Hauser, Aaron G. Schmidt, Lindsey R. Baden, Robert K. Ernst, Carly Dillen, Jingyou Yu, Aiquan Chang, Luuk Hilgers, Peter Paul Platenburg, Sirano Dhe-Paganon, Dan H. Barouch, Al Ozonoff, Ivan Zanoni, Matthew B. Frieman, David J. Dowling, Ofer Levy

**Affiliations:** 1 Precision Vaccines Program, Boston Children’s Hospital, Boston, MA USA; 2 Department of Pediatrics, Harvard Medical School, Boston, MA USA; 3 Division of Immunology, Boston Children’s Hospital, Boston, MA USA; 4 Department of Cancer Biology, Dana-Farber Cancer Institute, Boston, MA USA; 5 Department of Biological Chemistry and Molecular Pharmacology, Harvard Medical School, Boston, MA USA; 6 Department of Microbiology and Immunology, Center for Pathogen Research, University of Maryland School of Medicine, Baltimore, MD USA; 7 Research Computing Group, Boston Children’s Hospital, Boston, MA USA; 8 Ragon Institute of MGH, MIT, and Harvard, Cambridge, MA USA; 9 Department of Microbiology, Harvard Medical School, Boston, MA USA; 10 Department of Medicine, Brigham and Women’s Hospital, Boston, MA USA; 11 Department of Microbial Pathogenesis, University of Maryland School of Dentistry, Baltimore, MD USA; 12 Center for Virology and Vaccine Research, Beth Israel Deaconess Medical Center, Harvard Medical School, Boston, MA USA; 13 LiteVax B.V, Oss, The Netherlands; 14 Broad Institute of MIT & Harvard, Cambridge, MA USA; 15 Present Address: Generate Biomedicines, Cambridge, MA USA

**Keywords:** Adjuvants, Antibodies

Correction to: *npj Vaccines* 10.1038/s41541-023-00610-4, published online 14 February 2023

In this article Hyuk-Soo Seo, Timothy R. O’Meara, Marisa E. McGrath was incorrectly denoted as being one of the equally contributing authors. The original article has been corrected.

